# A simplified nomogram for predicting deep vein thrombosis: development in NHANES and retrospective single-center external validation

**DOI:** 10.1080/07853890.2026.2716932

**Published:** 2026-08-14

**Authors:** Zhenlin Gu, Yiyuan Zhang, Hongchao Zhu, Jing Huang

**Affiliations:** ^a^ Department of Vascular Surgery, The Affiliated Huaian No.1 People’s Hospital of Nanjing Medical University, Huaian, China; ^b^ Department of Nephrology, The Affiliated Huaian No.1 People’s Hospital of Nanjing Medical University, Huaian, China; ^c^ Department of Nuclear Medicine, The Affiliated Huaian No.1 People’s Hospital of Nanjing Medical University, Huaian, China; ^d^ Department of Radiation Oncology, The Affiliated Huaian No.1 People’s Hospital of Nanjing Medical University, Huaian, China

**Keywords:** Deep vein thrombosis, nomogram, risk prediction, NHANES, external validation, primary care, community screening

## Abstract

**Background:**

Deep vein thrombosis (DVT) is a major cardiovascular burden, and existing risk-stratification tools often rely on laboratory biomarkers, limiting their utility in primary care. We developed and externally validated a simplified, laboratory-free nomogram to predict DVT in community settings.

**Methods:**

The model was developed using 39,692 participants from NHANES(2005–2018; self-reported physician-diagnosed DVT) and externally validated in 3,136 hospitalized Chinese patients(2016–2023; ultrasound-confirmed DVT). Predictors were selected by LASSO and fitted using multivariable logistic regression incorporating the NHANES complex survey design. Performance was assessed by AUC, calibration (MAE), and decision curve analysis (DCA).

**Results:**

Four predictors were retained: age, smoking, physical activity, and diabetes. In development, the nomogram showed good discrimination (AUC, 0.816), comparable to XGBoost (0.820). Diabetes (OR, 2.84; 95% CI, 2.36–3.43) and ever-smoking (OR, 2.25; 95% CI, 1.92–2.65) were the strongest predictors. On external validation, discrimination was maintained (AUC, 0.792; 95% CI, 0.768–0.816) with good calibration (MAE, 0.022) and clinical net benefit across thresholds probabilities.

**Conclusions:**

This four-variable nomogram may help identify adults at higher risk of DVT in primary-care settings. It is intended as an exploratory screening aid, not a standalone diagnostic test or for suspected acute DVT. Prospective validation is required before clinical implementation.

## Introduction

Deep vein thrombosis (DVT) remains a formidable global health challenge, with an annual incidence of 1.0 to 2.0 per 1,000 individuals in Western populations [1] – a rate now rapidly escalating in Asia due to population aging and shifts toward sedentary lifestyles [2]. Venous thromboembolism is the third leading cause of cardiovascular mortality worldwide, and DVT – its principal precursor – is a major driver of pulmonary embolism and post-thrombotic syndrome [3]. Critically, DVT and pulmonary embolism (PE) are two clinical manifestations of a single pathological entity – venous thromboembolism (VTE). Approximately one-third of VTE cases present as PE, which carries a case-fatality rate of up to 10% and may manifest as sudden death without a diagnostic window. Because lower-extremity DVT is the principal source and precursor of most PE events, early identification of patients at elevated DVT risk offers an actionable upstream opportunity to interrupt progression toward fatal PE – particularly in primary care and community settings, where advanced PE diagnostics such as computed tomographic pulmonary angiography (CTPA) are often unavailable. Despite thromboprophylaxis efforts, the incidence of asymptomatic DVT in hospital settings ranges from 10% to 40%, representing a leading cause of preventable mortality [4]. Current clinical management relies heavily on established scores; however, their utility is often constrained by the low specificity of D-dimer assays, which frequently falls below 45% in elderly or comorbid patients – leading to an over-reliance on costly, negative imaging, which imposes a significant economic strain on healthcare systems and contradicts the principles of value-based care [5,6]. Importantly, these established pathways – which combine a clinical probability score with D-dimer testing and compression ultrasonography – are designed to diagnose or exclude DVT in patients with suspected acute disease, and are distinct from tools intended for risk prediction or screening in currently asymptomatic populations. In primary-care and resource-limited settings, where D-dimer testing and vascular imaging are frequently unavailable, a prediction tool based on routinely available clinical variables could help identify individuals at higher risk who warrant further assessment, without requiring laboratory testing at the point of contact. Several established instruments – such as the Wells, Caprini, and Padua scores – are widely used for DVT probability assessment or VTE risk stratification in clinical settings; however, they rely on acute clinical variables (e.g. localized tenderness or swelling, recent immobilization, or active malignancy) that are not captured in large population surveys such as NHANES, so a direct head-to-head comparison was not feasible in the present study, a point we revisit as a limitation. Furthermore, the generalizability of Western-derived models to diverse ethnic cohorts is increasingly questioned, with evidence suggesting a reduction in sensitivity of up to 15% in certain Asian populations, necessitating a more accessible and universally validated framework [7,8].

To address these limitations, the National Health and Nutrition Examination Survey (NHANES) provides a robust platform for epidemiological modeling, representing the diverse, non-institutionalized U.S. population [9]. This vast repository helps identify fundamental risk factors that extend beyond traditional laboratory-centric metrics. By distilling core clinical parameters from this large-scale dataset, it is possible to develop a predictive model that is both statistically rigorous and globally adaptable [10]. Although the resulting predictors – age, diabetes, smoking, and physical inactivity – are general cardiovascular–metabolic rather than VTE-specific risk factors, each maps onto a component of Virchow’s triad (endothelial dysfunction, hypercoagulability, and venous stasis), providing a plausible biological basis for their association with DVT at the population level. However, the cross-border generalizability of NHANES-derived models – particularly when applied to distinct clinical environments in China, where baseline metabolic profiles and lifestyle factors may differ significantly from those of the NHANES-based development cohort – remains largely unexplored.

The primary objective of this study was to develop and externally validate a simplified clinical nomogram to predict DVT [11]. Leveraging 39,692 participants from the NHANES database (2005–2018) for model development, we sought to identify a parsimonious set of clinical predictors that bypass the requirement for specialized laboratory markers. We then conducted rigorous external validation using an independent cohort of 3,136 patients from a high-volume tertiary hospital in China (2016–2023). Through this external validation, we aimed to bridge the gap between broad epidemiological surveillance and real-world clinical practice, providing a cost-effective tool based on routinely available clinical variables to support the early identification of individuals at higher DVT risk and, by extension, upstream prevention of VTE across heterogeneous healthcare settings.

## Methods

### Study population and data source

The development and internal validation of the predictive model utilized data from seven consecutive biennial cycles of the National Health and Nutrition Examination Survey (NHANES, 2005–2018). Conducted by the National Center for Health Statistics (NCHS), NHANES employs a complex, multistage, stratified, probability sampling design to provide a nationally representative assessment of the non-institutionalized US population [12]. From an initial pool of 70,190 participants, we excluded those younger than 20 years (*n* = 30,441) and those with incomplete Medical Examination Center sessions or missing data for the primary outcome – self-reported physician-diagnosed deep vein thrombosis (DVT) (*n* = 57). DVT status was ascertained *via* standardized questionnaires. This yielded a final development cohort of 39,692 participants. Missing covariates were addressed *via* Multiple Imputation by Chained Equations (MICE), and appropriate sampling weights were incorporated to ensure national representativeness. The NHANES protocol received approval from the NCHS Ethics Review Board, and all participants provided written informed consent.

**Figure 1.**
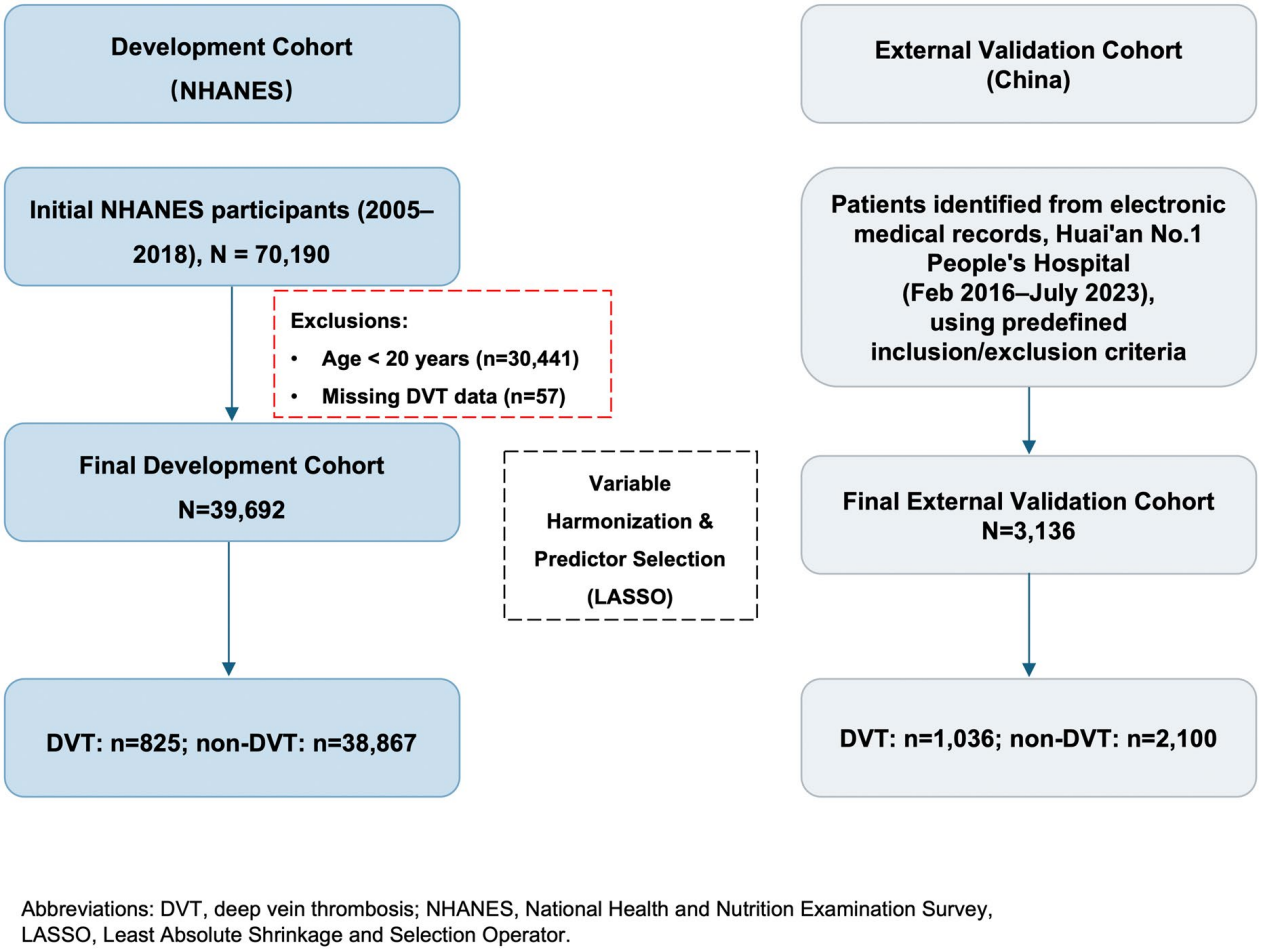
Study flowchart depicting participant selection and cohort establishment. The research framework integrated a development cohort from the National Health and Nutrition Examination Survey (NHANES) and an external validation cohort from a tertiary hospital in China. For the development cohort, NHANES participants (2005–2018) were initially screened (*N* = 70,190), with exclusions based on age (< 20 years, *n* = 30,441) and missing primary outcome (deep vein thrombosis [DVT]) status (*n* = 57), resulting in a final analytical cohort of 39,692 individuals (825 DVT and 38,867 non-DVT cases). LASSO regression was then employed within this development cohort to select optimal predictors. Concurrently, an external validation cohort was established by identifying patients from the electronic medical records of The Affiliated Huaian No.1 People’s Hospital of Nanjing Medical University between February 2016 and July 2023 using predefined inclusion and exclusion criteria. After variable harmonization, the final 4-variable model was validated in 3,136 patients (1,036 DVT and 2,100 non-DVT cases). **Abbreviations:** DVT, deep vein thrombosis; NHANES, National Health and Nutrition Examination Survey; LASSO, Least Absolute Shrinkage and Selection Operator.

To evaluate geographical and clinical generalizability, an independent clinical cohort was retrospectively established at The Affiliated Huaian No.1 People’s Hospital of Nanjing Medical University. We consecutively screened patients admitted between February 2016 and July 2023. Applying inclusion and exclusion criteria consistent with the development cohort, 3,136 participants were enrolled, comprising 1,036 patients with a primary diagnosis of DVT and 2,100 without DVT. In this clinical setting, DVT was diagnosed by compression ultrasonography (CUS) in all cases, which served as the diagnostic reference standard; contrast-enhanced computed tomography venography (CTV) was performed after admission in the DVT group to assess proximal thrombus extension into the pelvic and iliac veins rather than as the diagnostic reference. The DVT cases were admitted primarily for symptomatic lower-extremity DVT, whereas the 2,100 non-DVT participants were admitted for a range of non-thrombotic conditions – including orthopedic surgery, general abdominal surgery, and chronic internal-medicine illnesses – and had no clinical signs or symptoms of venous thromboembolism. In line with routine clinical practice and radiation-safety considerations, confirmatory imaging was not performed in these asymptomatic individuals. This study followed the TRIPOD reporting guideline [13] and was approved by the Institutional Review Board of The Affiliated Huaian No.1 People’s Hospital of Nanjing Medical University (No. KY-2025-092-01). Given the study’s retrospective nature and the use of de-identified data, the requirement for individual informed consent was waived. Detailed participant selection flow and baseline characteristics for both cohorts are presented in Figure 1 and Table 1, respectively.

**Table 1.** Baseline characteristics of the development and validation cohorts.

Characteristics	*Development (NHANES)*			Validation (Hospital)		
	Non-DVT (*n* = 38,867)	DVT (*n* = 825)	*P* value	Non-DVT (*n* = 2,100)	DVT (*n* = 1,036)	*P* value
**Age, years (mean ± SD)**	49.4 ± 18.0	65.5 ± 12.5	< .001	59.2 ± 12.6	60.7 ± 10.9	< .001
**BMI, kg/m² (mean ± SD)**	29.2 ± 7.0	29.0 ± 7.8	.663	23.6 ± 2.7	24.1 ± 3.2	.015
**Glucose, mg/dL (median [IQR])**	100.0 [93.0–111.0]	107.0 [97.0–121.0]	< .001	110.3 [93.1–128.2]	102.0 [90.0–118.0]	< .001
**Total Cholesterol, mg/dL**	190.0 [164.0–218.0]	183.0 [157.0–214.0]	< .001	178.0 [151.7–201.5]	192.0 [165.0–215.0]	< .001
**Triglycerides, mg/dL**	102.0 [71.0–150.0]	109.0 [78.0–164.0]	< .001	198.2 [121.9–268.4]	185.0 [130.0–245.0]	< .001
**SBP, mmHg (mean ± SD)**	124.3 ± 19.0	129.3 ± 19.4	< .001	127.9 ± 15.0	134.5 ± 16.2	< .001
**Diabetes Mellitus, n (%)**			< .001			< .001
Yes	4,898 (12.6)	223 (27.0)		227 (10.8)	168 (16.2)	
No	33,969 (87.4)	602 (73.0)		1,873 (89.2)	868 (83.8)	
**Smoking History, n (%)**			< .001			< .001
Yes	16,847 (43.4)	753 (91.4)		459 (21.9)	480 (46.3)	
No	21,993 (56.6)	71 (8.6)		1,641 (78.1)	556 (53.7)	
**Physical Activity, n (%)**			< .001			< .001
Yes	8,286 (21.3)	35 (4.2)		840 (40.0)	155 (15.0)	
No	30,579 (78.7)	790 (95.8)		1,260 (60.0)	881 (85.0)	

Note: Continuous variables are presented as mean ± SD or median [interquartile range], and categorical variables as n (%). Continuous variables were compared using the Student’s t-test or Mann–Whitney U test, and categorical variables using the chi-square or Fisher’s exact test, as appropriate. A two-sided *p* < .05 was considered statistically significant. Abbreviations: BMI, body mass index; DVT, deep vein thrombosis; IQR, interquartile range; NHANES, National Health and Nutrition Examination Survey; SBP, systolic blood pressure; SD, standard deviation.

### Variable definition and predictor selection

To ensure cross-cohort comparability, all variables were harmonized before analysis. The primary outcome was a physician-confirmed diagnosis of deep vein thrombosis (DVT). In the NHANES cohort, DVT was ascertained *via* standardized questionnaires, and in the validation cohort, it was diagnosed by compression ultrasonography (CUS). Contrast-enhanced computed tomography venography (CTV) was used to assess proximal thrombus extension rather than as the diagnostic reference. Four core clinical predictors were operationally defined: (1) Age was treated as a continuous variable; (2) Diabetes mellitus was defined by a documented physician diagnosis or use of hypoglycemic agents; (3) Smoking status was dichotomized into ‘Ever-smokers’ (those with a documented history of habitual smoking) and ‘Never-smokers’; (4) Physical activity was assessed as a binary variable, denoting participation in regular moderate-to-vigorous exercise versus a sedentary lifestyle.

The candidate pool comprised 12 variables routinely available in primary care without specialized testing: age, sex, body mass index, systolic and diastolic blood pressure, diabetes status, smoking history, physical activity, alcohol consumption, total cholesterol, triglycerides, and fasting glucose. These candidates were specified a priori based on clinical accessibility, support from prior literature, and a prespecified missingness threshold (variables with >50% missingness were excluded). To refine the model and minimize overfitting, Least Absolute Shrinkage and Selection Operator (LASSO) regression was employed for predictor selection [14]. To respect the NHANES complex survey design, predictor selection used design-adjusted LASSO (R surveycv with glmnet), which accounts for sampling weights, strata (SDMVSTRA), and primary sampling units during 10-fold cross-validation. Variables with non-zero coefficients at the 1-standard-error (1-SE) criterion were retained (age, diabetes, smoking history, and physical activity) and incorporated into the final multivariable logistic regression to construct the predictive nomogram.

### Model development and visualization

A multivariable logistic regression model was constructed, incorporating the four independent predictors identified *via* LASSO. The strength of the association between each predictor and DVT risk was quantified using odds ratios (ORs) and their corresponding 95% confidence intervals (CIs). To translate the mathematical model into a user-friendly clinical tool, we developed a predictive nomogram using the rms package [11] in R software (version 4.4.1; R Foundation for Statistical Computing). This visualization tool assigns a weighted score to each variable, enabling calculation of an individual’s cumulative probability of DVT based on their clinical profile. The total score, derived by summing individual points, corresponds to a specific risk estimate on the nomogram’s scale, thereby facilitating individualized risk prediction.

### Model evaluation, validation, and comparison

The predictive performance of the nomogram was evaluated and compared with a machine-learning model, Extreme Gradient Boosting (XGBoost), to benchmark its discriminatory power. The XGBoost model was implemented in R using the XGBoost package, with hyperparameters optimized *via* grid search. Discrimination was quantified using the area under the receiver operating characteristic (ROC) curve (AUC). Calibration was assessed using calibration plots and the calibration slope, calibration intercept, and mean absolute error (MAE) to evaluate concordance between predicted probabilities and observed outcomes. Furthermore, Decision Curve Analysis (DCA) was performed to determine the model’s clinical utility [15] by calculating net benefits across a range of threshold probabilities. This framework enabled comparison of the nomogram with more complex algorithms and evaluation of its performance for external DVT risk prediction.

### Statistical analysis

Continuous variables were expressed as means with standard deviations (SD) or medians with interquartile ranges (IQR), depending on their distribution, and compared using the Student’s t-test or Mann-Whitney U test. Categorical variables were presented as frequencies and percentages, with comparisons performed using the Chi-square test or Fisher’s exact test. In the NHANES development cohort (*n* = 39,692), missingness was minimal for all predictors: age, 0% (*n* = 0); diabetes status, 0% (*n* = 0); smoking history, 0.07% (*n* = 28); and physical activity, <0.01% (*n* = 2). Missing values were handled using Multiple Imputation by Chained Equations (MICE; R mice package); five datasets were imputed and pooled using Rubin’s rules. Given the negligible missingness, imputation had no material effect on the estimates, and the results were essentially identical to those from a complete-case analysis. In the validation cohort, missingness was <1% for all retained variables, and a complete-case analysis was used. For the NHANES development cohort, all analyses incorporated the complex survey design, including the examination weights (WTMEC2YR), strata (SDMVSTRA), and primary sampling units; the final model coefficients and odds ratios were estimated using survey-weighted multivariable logistic regression (R survey package). External validation applied the original NHANES-derived coefficients and intercept to the Chinese cohort without recalibration, intercept adjustment, or refitting, and discrimination and calibration were assessed in this fixed model. Model development and validation were conducted in R (version 4.4.1), using the survey, surveycv, glmnet, mice, rms, and xgboost packages. All tests were two-sided, and a *p* < .05 was considered statistically significant.

## Results

### Baseline characteristics and cohort profiles

A total of 39,692 participants from the NHANES database (Development Cohort) and 3,136 patients from the Chinese clinical registry (External Validation Cohort) were included in the final analysis. The comprehensive baseline clinical and demographic characteristics of both cohorts, stratified by the presence of deep vein thrombosis (DVT), are summarized in Table 1. The selection process for both populations is illustrated in the study flowchart (Figure 1).

In the development cohort, individuals with DVT (*n* = 825) were significantly older than those without (mean [SD] age, 65.48 [12.49] vs 49.35 [17.95] years; *p* < .001) and had a markedly higher burden of metabolic comorbidities. Specifically, the DVT group had a higher prevalence of diabetes mellitus (27.0% vs 12.6%) and a more extensive smoking history (91.4% vs 43.4%). Furthermore, sedentary lifestyles were disproportionately prevalent among DVT patients, with only 4.2% reporting regular physical activity compared with 21.3% in the control group (all *p* < .001).

Similar clinical associations were corroborated in the external validation cohort. Notably, the DVT group (*n* = 1,036) had significantly higher systolic blood pressure and a higher prevalence of diabetes mellitus (16.2% vs. 10.8% in the non-DVT group) and smoking history (46.3% vs. 21.9%). Despite inherent baseline disparities between the U.S. population-based survey and the Chinese clinical cohort – particularly in BMI (mean, 29.15 vs. 23.63 kg/m^2^) and lipid profiles – the core predictors (advanced age, diabetes, smoking, and physical inactivity) were consistently associated with DVT across both geographical and clinical settings.

### Predictor selection and nomogram construction

**Figure 2.**
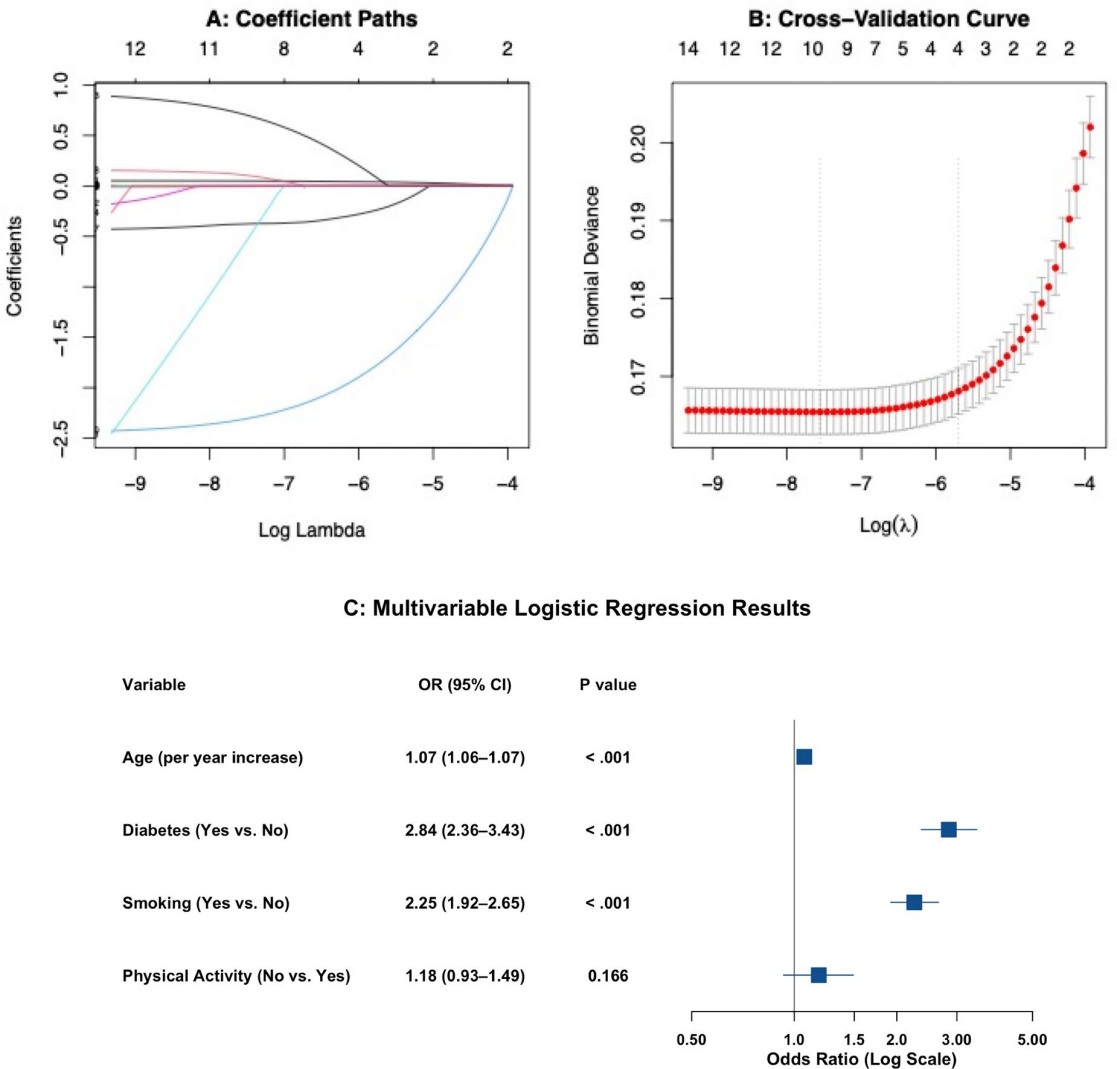
Variable selection and multivariable logistic regression analysis for DVT risk factors. The figure illustrates the process of predictor selection and model construction. (A) LASSO coefficient paths. Each curve represents the coefficient of a potential predictor as a function of the L1 penalty (Log Lambda). As the penalty increases, non-essential variables are shrunk to zero, mitigating overfitting. (B) LASSO parameter selection *via* cross-validation. The binomial deviance is plotted against Log(λ). The two vertical dashed lines indicate the λ at minimum deviance and the 1-standard-error (1-SE) criterion, with the latter used to select a parsimonious set of four final predictors. (C) Forest plot of multivariable logistic regression results. Odds ratios (ORs) and 95% confidence intervals (CIs) are shown for the four variables selected *via* LASSO. Age (per year), diabetes, and smoking history were significantly and independently associated with DVT risk (all *p* <.001), whereas physical activity did not reach statistical significance (*p* =.166). **Abbreviations:** CI, confidence interval; DVT, deep vein thrombosis; LASSO, Least Absolute Shrinkage and Selection Operator; OR, odds ratio.

To ensure a parsimonious model and prevent overfitting, we used LASSO regression to screen candidate predictors in the development cohort (Figure 2A,B). In the final survey-weighted multivariable logistic regression model, age, diabetes, and smoking were significantly and independently associated with DVT (all *p* < .001). Although physical activity was retained by LASSO, it did not reach statistical significance (Figure 2C; Table 2). Diabetes (OR, 2.84; 95% CI, 2.36–3.43) and ever-smoking (OR, 2.25; 95% CI, 1.92–2.65) were the strongest independent risk factors, and each additional year of age was associated with a modest increase in risk (OR, 1.07; 95% CI, 1.06–1.07). Physical inactivity showed a non-significant association after adjustment (OR, 1.18; 95% CI, 0.93–1.49; *p* = .166).

**Table 2.** Multivariable survey-weighted logistic regression analysis of risk factors for DVT.

Variable	Beta	S.E.	OR (95% CI)	*P* value
**Age (per year increase)**	0.0659	0.0024	1.07 (1.06–1.07)	< .001
**Diabetes (Yes vs. No)**	1.0447	0.0944	2.84 (2.36–3.43)	< .001
**Smoking (Yes vs. No)**	0.8120	0.0809	2.25 (1.92–2.65)	< .001
**Physical Activity (No vs. Yes)**	0.1640	0.1173	1.18 (0.93–1.49)	0.166

Note: Estimates were derived from a multivariable survey-weighted logistic regression model that accounted for the NHANES complex survey design; the four variables shown constitute the final parsimonious model selected by LASSO regression. Abbreviations: Beta, regression coefficient; CI, confidence interval; OR, odds ratio; S.E., standard error.

**Figure 3.**
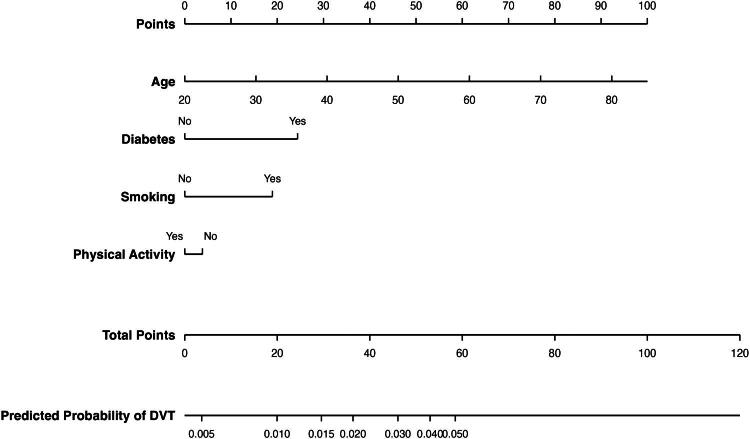
Predictive nomogram for individualized DVT risk assessment. The nomogram was constructed based on the four independent predictors identified in the multivariable logistic regression model from the NHANES development cohort. To estimate the risk of deep vein thrombosis (DVT), a vertical line is drawn upward from each variable value (Age, Diabetes Mellitus, Smoking Status, and Physical Activity) to the ‘Points’ scale to obtain the score for each factor. The sum of these individual scores is then located on the ‘Total Points’ axis. Finally, a vertical line is drawn downward to the ‘Predicted Probability of DVT’ axis to determine the individualized risk. Advanced age contributes the most to the cumulative risk score, followed by diabetes, smoking, and physical inactivity. **Abbreviations:** DVT, deep vein thrombosis; NHANES, National Health and Nutrition Examination Survey.

To facilitate individualized risk assessment, we integrated these predictors into a visual nomogram (Figure 3). Within this framework, each variable is assigned points proportional to its adjusted association with DVT, with older age, diabetes, and smoking contributing the most to the total score. By summing the scores across these four dimensions, clinicians can derive a total point value and map it to a predicted probability of DVT, thereby supporting individualized risk estimation.

### Model performance in the development cohort

**Figure 4.**
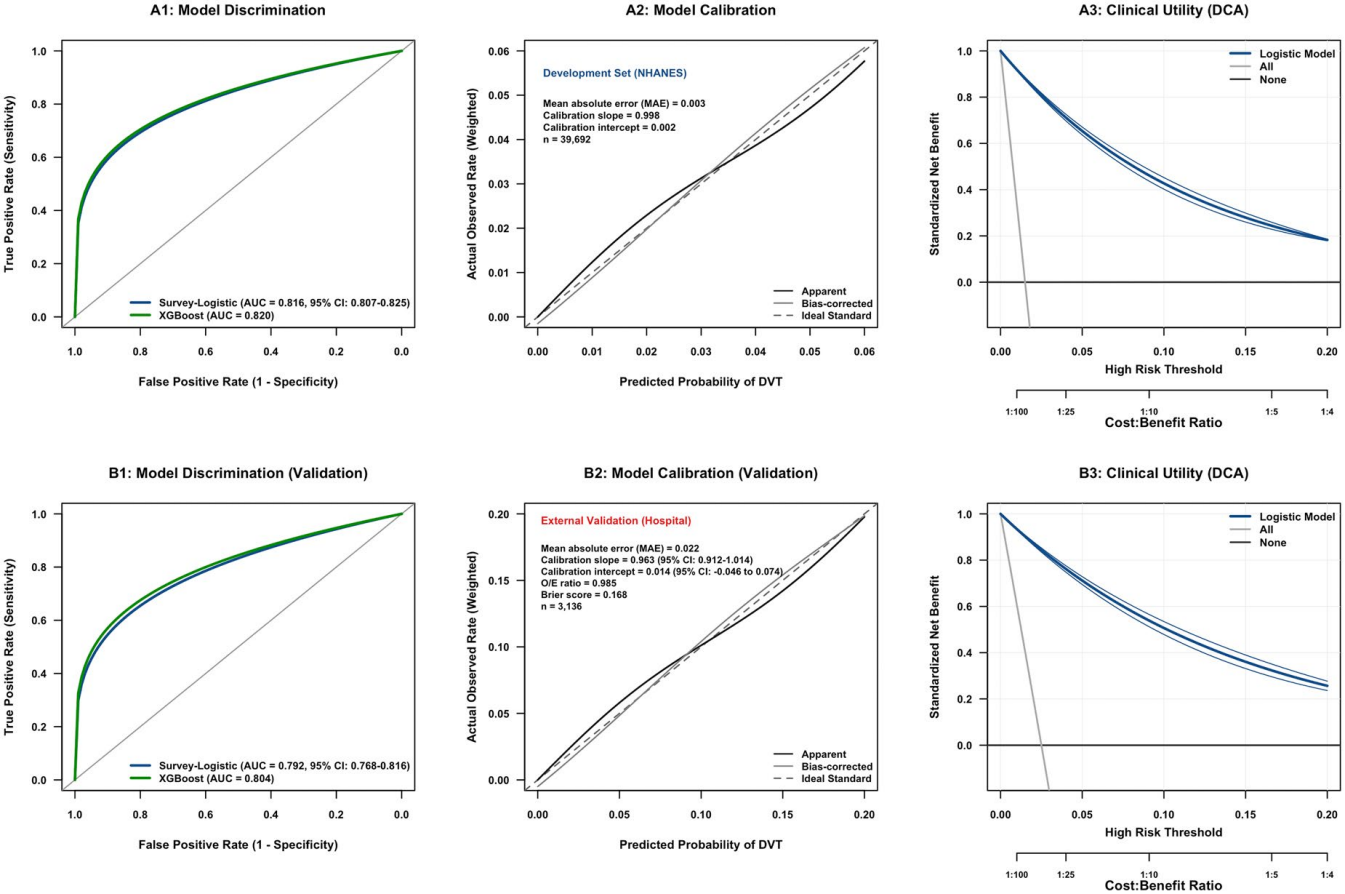
Performance evaluation of the DVT prediction models in the development and external validation cohorts. Development cohort (NHANES): (A1) ROC curves illustrating the discrimination of the logistic and XGBoost models, with the logistic model achieving an AUC of 0.816 (XGBoost, 0.820); (A2) Calibration plot for the logistic model, showing close agreement between predicted and observed DVT probabilities (MAE = 0.003; calibration slope, 0.998; intercept, 0.002); (A3) Decision curve analysis (DCA) demonstrating net clinical benefit across a wide range of threshold probabilities. External validation cohort (The Affiliated Huaian No.1 People’s Hospital of Nanjing Medical University): (B1) ROC curves in the clinical cohort (Logistic AUC = 0.792; XGBoost = 0.804); (B2) Calibration plot for the external cohort, indicating a mean absolute error (MAE) of 0.022, a calibration slope of 0.963, and an intercept of 0.014; (B3) DCA confirming the clinical utility of the predictive model in a real-world hospital setting. **Abbreviations:** AUC, area under the curve; DCA, decision curve analysis; DVT, deep vein thrombosis; MAE, mean absolute error; NHANES, National Health and Nutrition Examination Survey; ROC, receiver operating characteristic.

The nomogram’s predictive performance was evaluated in the NHANES development cohort. The model demonstrated good discrimination, with an AUC of 0.816 (95% CI, 0.807–0.825). This was comparable to a more complex machine-learning model (XGBoost AUC = 0.820), suggesting that the parsimonious four-variable model captures most of the available predictive signal (Figure 4A1). The model also showed good calibration in the development cohort (Figure 4A2), with predicted and observed probabilities closely aligned (MAE = 0.003). Decision Curve Analysis (DCA) supported the model’s clinical utility, showing net benefit across a broad range of threshold probabilities (Figure 4A3).

### External validation and clinical utility

The nomogram’s generalizability was tested using the independent, hospital-based validation cohort (*n* = 3,136). In this clinical setting, the nomogram achieved an AUC of 0.792 (95% CI, 0.768–0.816) (Figure 4B1). Discrimination was comparable to that of a more complex model (XGBoost AUC = 0.804), and we selected the logistic-based nomogram for its greater interpretability. Discrimination was largely preserved relative to the development cohort (0.816 to 0.792), consistent with the demographic and clinical differences between the U.S. community population and the Chinese inpatient population.

Decision Curve Analysis (DCA) confirmed the tool’s clinical value (Figure 4B3): across a broad range of threshold probabilities, the nomogram yielded a higher net benefit than either the ‘screen-all’ or ‘screen-none’ strategies. The calibration curve for the external validation cohort (Figure 4B2) showed good agreement, with a calibration slope of 0.963 (95% CI, 0.912–1.014), an intercept of 0.014 (95% CI, −0.046 to 0.074), an observed-to-expected ratio of 0.985 (95% CI, 0.943–1.028), a mean absolute error of 0.022, and a Brier score of 0.168.

At a pre-specified probability threshold of 12.5% (the value that maximized the Youden index in the development cohort), the nomogram achieved a sensitivity of 82.4% (95% CI, 80.0–84.7), a specificity of 62.8% (95% CI, 60.7–64.9), a positive predictive value of 52.2% (95% CI, 49.8–54.6), and a negative predictive value of 88.1% (95% CI, 86.4–89.7) in the external validation cohort. The relatively high sensitivity and negative predictive value are consistent with the model’s intended use as an upstream screening aid.

## Discussion

In this large-scale, multi-cohort study, we developed and externally validated a parsimonious nomogram to predict deep vein thrombosis (DVT) [16,17]. By leveraging the NHANES database (*N* = 39,692) and an independent Chinese clinical cohort (*N* = 3,136), a four-variable model incorporating age, smoking status, physical activity, and diabetes showed consistent predictive performance across two distinct geographical and clinical settings [12,18]. Our model achieved an AUC of 0.816 in the development cohort, performing comparably to a more complex machine-learning model such as XGBoost (AUC 0.820) [19]. This suggests that, for this outcome, clinical parsimony does not substantially compromise discrimination relative to more complex algorithms [13,20].

The predictive utility of this model is grounded in the biological plausibility of its components [21]. In the multivariable analysis, diabetes (OR 2.84; 95% CI, 2.36–3.43) and ever-smoking (OR 2.25; 95% CI, 1.92–2.65) were the strongest independent risk factors, consistent with hyperglycemia-related increases in blood viscosity and a smoking-induced pro-thrombotic state mediated by systemic inflammation and endothelial injury [2,22]. Advancing age was likewise independently associated with DVT (OR 1.07 per year; 95% CI, 1.06–1.07), in keeping with age-related venous valvular degeneration. Although physical inactivity was substantially more common among patients with DVT in both cohorts at the descriptive level – consistent with the role of the skeletal-muscle pump in mitigating venous stasis, a component of Virchow’s triad [1,4] – its independent association did not reach statistical significance after adjustment (OR 1.18 for inactivity; 95% CI, 0.93–1.49; *p* = 0.166). It was nonetheless retained by the LASSO procedure for its contribution to overall predictive performance [23,24].

A key consideration in our study was the transition from community-based survey data to real-world clinical validation [25]. Discrimination was largely preserved across these disparate settings, with the AUC decreasing modestly from 0.816 in the development cohort to 0.792 in the external validation cohort, accompanied by acceptable calibration (mean absolute error, 0.022) [26]. This stability is notable given the differing epidemiological profiles of the two cohorts – for example, the prevalence of diabetes among patients with DVT was 16.2% in the validation cohort compared with 27.0% in NHANES. The validation AUC of 0.792 also exceeded the performance previously reported for the Wells score in comparable Chinese settings (AUC 0.65–0.72); nonetheless, an AUC of 0.792 represents moderate discrimination, and this consistency supports transportability between the two cohorts studied rather than definitive clinical accuracy [2,27,28].

Although XGBoost provided a marginal improvement in discrimination, we ultimately prioritized multivariable logistic regression for the final nomogram [29]. This choice addresses the imperative for clinical interpretability; unlike ‘black-box’ machine learning models, the logistic-based nomogram provides a transparent, bedside-ready interface that allows clinicians to visualize individual risk contributions without specialized software [11,30]. This balance between performance and pragmatism supports its practical use in primary care [31].

Decision Curve Analysis (DCA) indicated a net clinical benefit across a range of threshold probabilities [15]. By relying only on routinely available variables, the tool may help frontline clinicians in resource-limited settings identify individuals at higher risk who warrant further assessment [32].

Whether this translates into more efficient use of diagnostic resources or improved outcomes remains to be evaluated in prospective studies. Our findings should be interpreted within the broader context of VTE. DVT and PE are two manifestations of the same disease process, and PE – accounting for roughly one-third of VTE presentations – carries a case-fatality rate of up to 10%. Our decision to target DVT reflects a deliberate strategy of pre-emptive risk stratification at the source: because lower-extremity DVT is the principal precursor of most PE events, identifying patients at elevated risk of DVT provides an upstream window for lower-limb venous ultrasonography and mechanical or pharmacological thromboprophylaxis before a potentially fatal PE develops. This upstream orientation is particularly valuable in resource-limited community settings, where PE-specific diagnostics such as CTPA are typically unavailable and acute PE frequently presents as sudden death. Our nomogram should therefore be regarded as an exploratory, upstream preventive tool within the VTE continuum rather than a stand-alone predictor of PE.

Several limitations warrant consideration. First, the dataset’s retrospective nature may introduce selection bias. Second, reliance on self-reported lifestyle data in NHANES may lead to minor classification discrepancies. Third, DVT in the development cohort was ascertained by self-reported physician diagnosis rather than imaging, which may introduce outcome misclassification. Together with imaging-confirmed ascertainment in the validation cohort, this difference in outcome definition may affect the model’s calibration and transportability. Fourth, candidate variables were restricted to general cardiovascular–metabolic factors and did not include several established VTE-specific risk factors – such as active malignancy, hormonal exposure, prolonged immobilization, and inflammatory disease – so the nomogram is intended as an accessible screening aid rather than a substitute for established VTE risk assessment (e.g. the Caprini or Wells scores); the small number of predictors also precluded a formal evaluation of interaction effects, which we identify as an avenue for future work. Fifth, the cross-sectional design of NHANES precludes causal inference and, as a survey of the non-institutionalized population, may under-represent critically ill or institutionalized individuals; residual confounding from unmeasured factors cannot be excluded. Sixth, because confirmatory imaging was not performed in asymptomatic control participants, a small number of occult DVT cases may have remained undetected in the non-DVT group, potentially resulting in minor outcome misclassification. Seventh, external validation was performed in a single hospital-based cohort and does not establish applicability to primary-care or community screening populations, in which disease prevalence, case mix, and pretest probability differ; prospective validation in the intended primary-care and community settings, ideally across multiple centers, is required before clinical use. Finally, our model targets DVT as the upstream presentation of VTE and was not designed to predict isolated PE directly; prospective studies incorporating PE outcomes are warranted to evaluate its performance across the full spectrum of VTE.

## Conclusion

In conclusion, we developed and externally validated a parsimonious four-variable nomogram – based on age, smoking, diabetes, and physical activity – that links large-scale epidemiological data with a real-world clinical cohort. The model offers a simplified, laboratory-free approach to DVT risk prediction and is intended as an exploratory screening aid to support the early identification of higher-risk individuals in primary-care and community settings, rather than as a stand-alone diagnostic test or a tool to direct thromboprophylaxis or specialist referral. In its current form, the model should be regarded as hypothesis-generating rather than ready for clinical implementation. Before it can be recommended for practice, it requires prospective validation and direct, head-to-head comparison with established risk-assessment and diagnostic algorithms (e.g. the Wells, Caprini, or Padua scores), ideally in multi-center studies conducted in the intended primary-care and community settings.

## Data Availability

The data supporting the findings of this study are available from two sources. The development cohort data are publicly available from the National Health and Nutrition Examination Survey (NHANES) repository (https://www.cdc.gov/nchs/nhanes/index.htm). The external validation data from The Affiliated Huaian No. 1 People’s Hospital of Nanjing Medical University are not publicly available due to institutional restrictions and patient privacy concerns but are available from the corresponding author upon reasonable request.

## References

[1] Wendelboe AM, Raskob GE. Global burden of thrombosis: epidemiologic Aspects. Circ Res. 2016;118(9):1340–1347. doi: 10.1161/CIRCRESAHA.115.306841. 27126645

[2] Zhang Z, Lei J, Shao X, et al. Trends in hospitalization and in-hospital mortality from VTE, 2007 to 2016, in China. Chest. 2019;155(2):342–353. doi: 10.1016/j.chest.2018.10.040. 30419233

[3] Raskob GE, Angchaisuksiri P, Blanco AN, et al. Thrombosis: a major contributor to global disease burden. Semin Thromb Hemost. 2014;40(7):724–735. doi: 10.1055/s-0034-1390325. 25302681

[4] Kahn SR, Comerota AJ, Cushman M, et al. The postthrombotic syndrome: evidence-based prevention, diagnosis, and treatment strategies: a scientific statement from the American heart association. Circulation. 2014;130(18):1636–1661. doi: 10.1161/CIR.0000000000000130. 25246013

[5] Wells PS, Anderson DR, Rodger M, et al. Evaluation of D-dimer in the diagnosis of suspected deep-vein thrombosis. N Engl J Med. 2003;349(13):1227–1235. doi: 10.1056/NEJMoa023153. 14507948

[6] Schouten HJ, Geersing GJ, Koek HL, et al. Diagnostic accuracy of conventional or age adjusted D-dimer cut-off values in older patients with suspected venous thromboembolism: Systematic review and meta-analysis. BMJ (Online). 2013 doi: 10.1136/BMJ.F2492. PMC3643284 23645857

[7] Gould MK, Garcia DA, Wren SM, et al. Prevention of VTE in nonorthopedic surgical patients. Antithrombotic therapy and prevention of thrombosis, 9th ed: american College of Chest Physicians evidence-based clinical practice guidelines. Chest. 2012;141(2 Suppl):e227S–e277S. doi: 10.1378/chest.11-2297. 22315263 PMC3278061

[8] Kahn SR, Lim W, Dunn AS, et al. Prevention of VTE in nonsurgical patients. Antithrombotic therapy and prevention of thrombosis, 9th ed: american College of Chest Physicians evidence-based clinical practice guidelines. Chest. 2012;141(2 Suppl):e195S–e226S. doi: 10.1378/chest.11-2296. 22315261 PMC3278052

[9] National Research Council (US) Committee on Evaluation of Food Consumption Surveys. (1984). The national health and nutrition examination survey.

[10] Collins GS, Reitsma JB, Altman DG, et al. Transparent reporting of a multivariable prediction model for individual prognosis or diagnosis (TRIPOD): the TRIPOD Statement. BMC Med. 2015;13(1):1. doi: 10.1186/s12916-014-0241-z. 25563062 PMC4284921

[11] Iasonos A, Schrag D, Raj GV, et al. How to build and interpret a nomogram for cancer prognosis. J Clin Oncol. 2008;26(8):1364–1370. doi: 10.1200/JCO.2007.12.9791. 18323559

[12] Wang L, Li X, Wang Z, et al. Trends in prevalence of diabetes and control of risk factors in diabetes among US adults, 1999-2018. JAMA. 2021;326(8):1–13. doi: 10.1001/jama.2021.9883. PMC8233946 34170288

[13] Collins GS, Reitsma JB, Altman DG, et al. Transparent reporting of a multivariable prediction model for individual prognosis or diagnosis (TRIPOD): the TRIPOD statement. BMJ. 2015;350(jan07 4):g7594–g7594. doi: 10.1136/bmj.g7594. 25569120

[14] Sauerbrei W, Perperoglou A, Schmid M, et al. State of the art in selection of variables and functional forms in multivariable analysis—outstanding issues. Diagn Progn Res. 2020;4:3. doi: 10.1186/s41512-020-00074-3. 32266321 PMC7114804

[15] Vickers AJ, Elkin EB. Decision curve analysis: a novel method for evaluating prediction models. Med Decis Making. 2006;26(6):565–574. doi: 10.1177/0272989X06295361. 17099194 PMC2577036

[16] Fleming KA, Horton S, Wilson ML, et al. The Lancet Commission on diagnostics: transforming access to diagnostics. Lancet. 2021;398(10315):1997–2050. doi: 10.1016/S0140-6736(21)00673-5. 34626542 PMC8494468

[17] Topol EJ. High-performance medicine: the convergence of human and artificial intelligence. Nat Med. 2019;25(1)2019 25:1 :44–56. doi: 10.1038/s41591-018-0300-7. 30617339

[18] Alba AC, Agoritsas T, Walsh M, et al. Discrimination and calibration of clinical prediction models: users’ guides to the medical literature. JAMA - Journal of the American Medical Association. 2017;318(14):1377–1384. doi: 10.1001/jama.2017.12126. 29049590

[19] Rajkomar A, Oren E, Chen K, et al. Scalable and accurate deep learning with electronic health records. NPJ Digit Med. 2018;1(1):18. doi: 10.1038/s41746-018-0029-1. 31304302 PMC6550175

[20] Steyerberg EW, Harrell FE, Borsboom GJJM, et al. Internal validation of predictive models: efficiency of some procedures for logistic regression analysis. J Clin Epidemiol. 2001;54(8):774–781. doi: 10.1016/s0895-4356(01)00341-9. 11470385

[21] Wolberg AS, Rosendaal FR, Weitz JI, et al. Venous thrombosis. Nat Rev Dis Primers. 2015;1(1):15006. doi: 10.1038/nrdp.2015.6. 27189130

[22] Messner B, Bernhard D. Smoking and cardiovascular disease: mechanisms of endothelial dysfunction and early atherogenesis. Arterioscler Thromb Vasc Biol. 2014;34(3):509–515. doi: 10.1161/ATVBAHA.113.300156. 24554606

[23] Xu Y, Wang L, He J, et al. Prevalence and control of diabetes in Chinese adults. JAMA. 2013;310(9):948–959. doi: 10.1001/jama.2013.168118. 24002281

[24] Goldhaber SZ. Risk factors for venous thromboembolism. J Am Coll Cardiol. 2010;56(1):1–7. doi: 10.1016/j.jacc.2010.01.057. 20620709

[25] Collins GS, Reitsma JB, Altman DG, et al. Transparent reporting of a multivariable prediction model for individual prognosis or diagnosis (TRIPOD): the TRIPOD statement. Ann Intern Med. 2015;162(1):55–63. doi: 10.7326/M14-0697. 25560714

[26] Zhen K, Tao Y, Xia L, et al. Epidemiology of pulmonary embolism in China, 2021: a nationwide hospital-based study. Lancet Reg Health West Pac. 2025;54:101258. doi: 10.1016/j.lanwpc.2024.101258. 39759425 PMC11699474

[27] Wang C, Zhai ZG, Shen YH. Challenges facing venous thromboembolism in China: more public awareness and research needed. Chin Med J (Engl). 2010;123:387–389. 20193473

[28] Tang L, Hu Y, Min M, et al. Comparisons of clinical scoring systems among suspected pulmonary embolism patients presenting to emergency department. Health Sci Rep. 2024;7(8):e70003. doi: 10.1002/hsr2.70003. 39170892 PMC11335811

[29] Lundberg SM, Erion G, Chen H, et al. From local explanations to global understanding with explainable AI for trees. Nat Mach Intell. 2020;2(1):56–67. doi: 10.1038/s42256-019-0138-9. 32607472 PMC7326367

[30] Shmueli G. To explain or to predict? Statist Sci. 2010;25(3):289–310. doi: 10.1214/10-STS330.

[31] Khan F, Tritschler T, Kahn SR, et al. Venous thromboembolism. Lancet. 2021;398(10294):64–77. doi: 10.1016/S0140-6736(20)32658-1. 33984268

[32] Van Calster B, Wynants L, Verbeek JFM, et al. Reporting and Interpreting Decision Curve Analysis: A Guide for Investigators. Eur Urol. 2018;74(6):796–804. doi: 10.1016/j.eururo.2018.08.038. 30241973 PMC6261531

